# Supplementary figures and images for: Dinoroseobacter shibae Outer Membrane Vesicles Are Enriched for the Chromosome Dimer Resolution Site dif
Source: mSystems. 2021 Jan 12;6(1):e00693-20. doi: 10.1128/mSystems.00693-20 (PMC7901474; doi:10.1128/mSystems.00693-20)

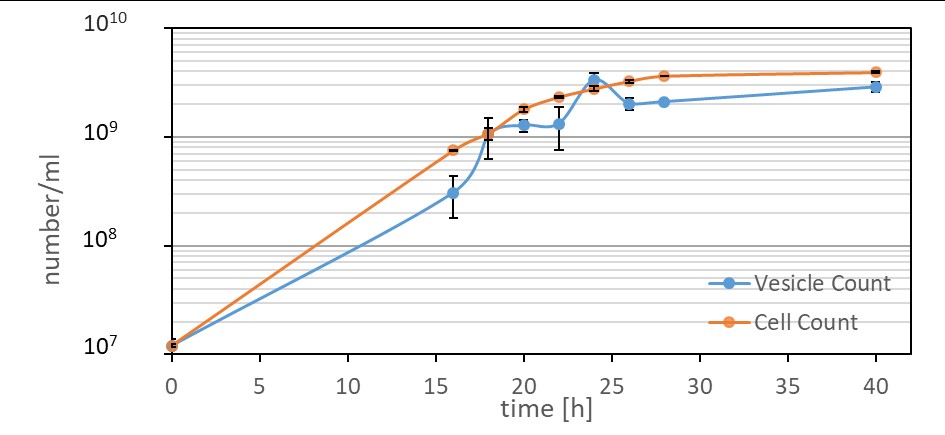

Supplement: FIG S1 [file mSystems.00693-20_sf001.jpg]

**Figure S2A**

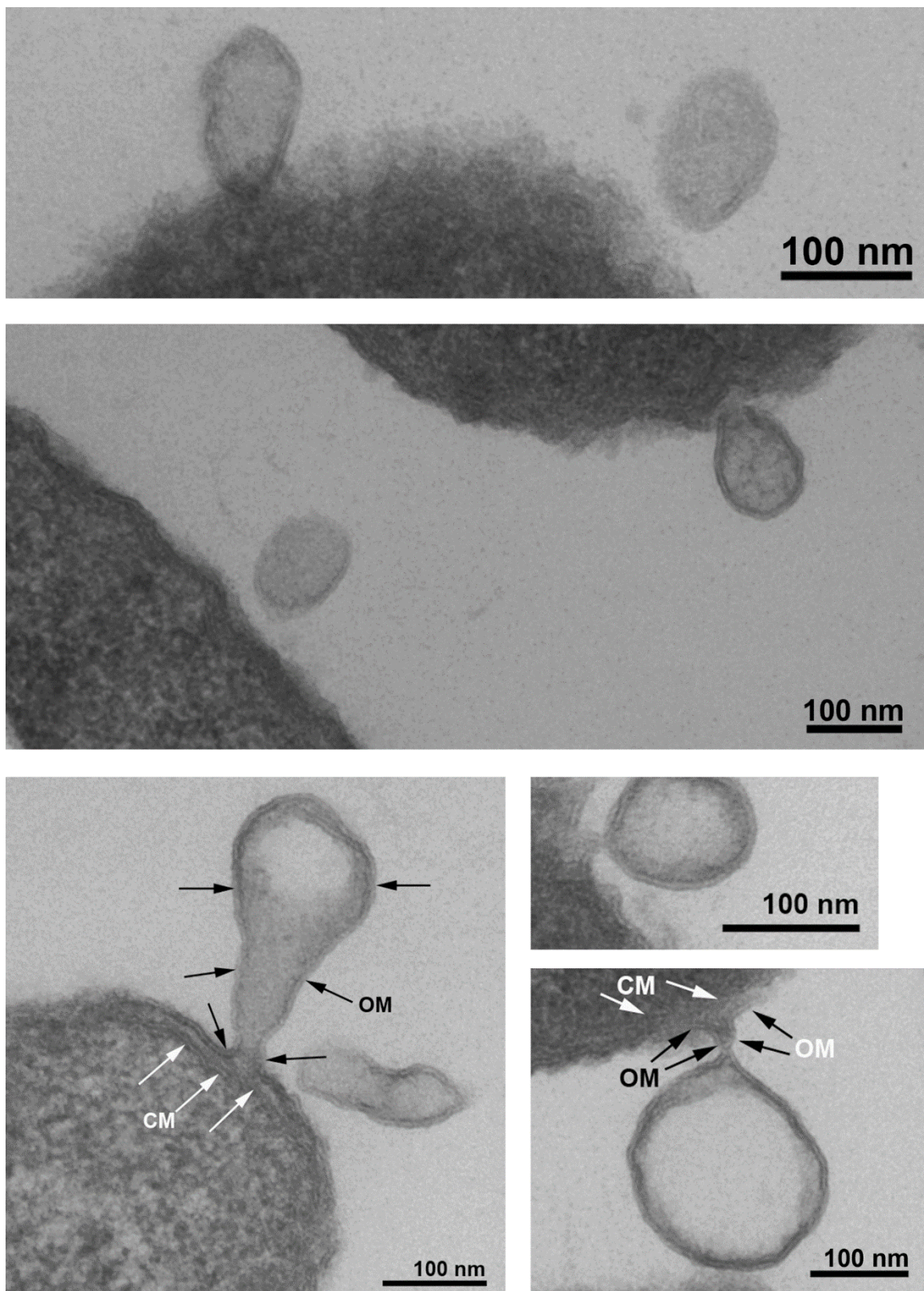

**Figure S2B.**

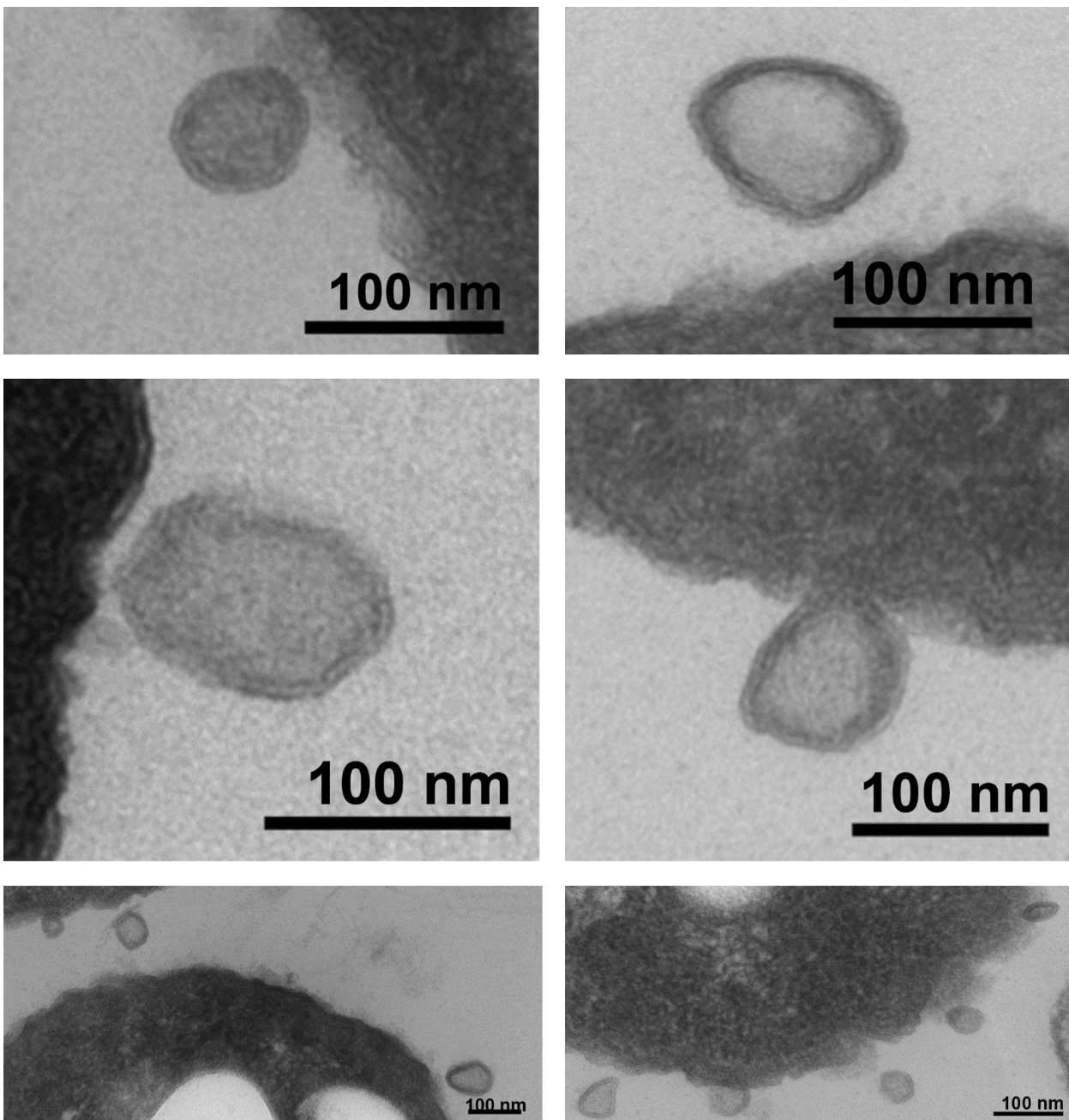

**Figure S2C**

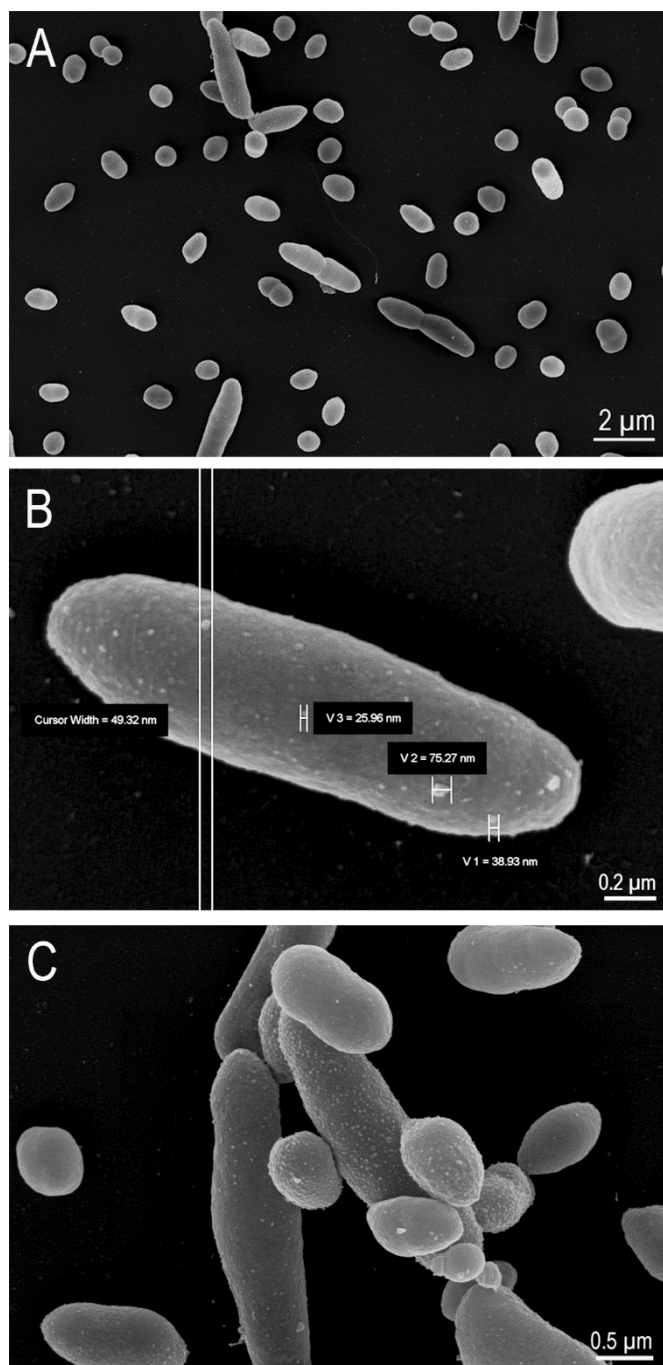

Supplement: FIG S2 [file mSystems.00693-20_sf002.pdf]

**Figure S3A**

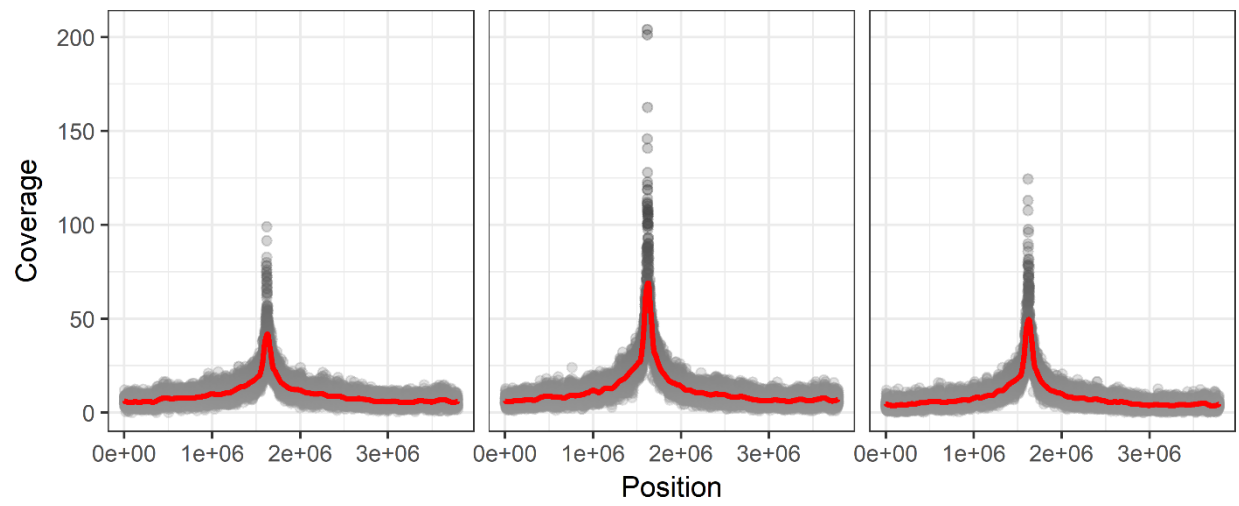

Figure S3B

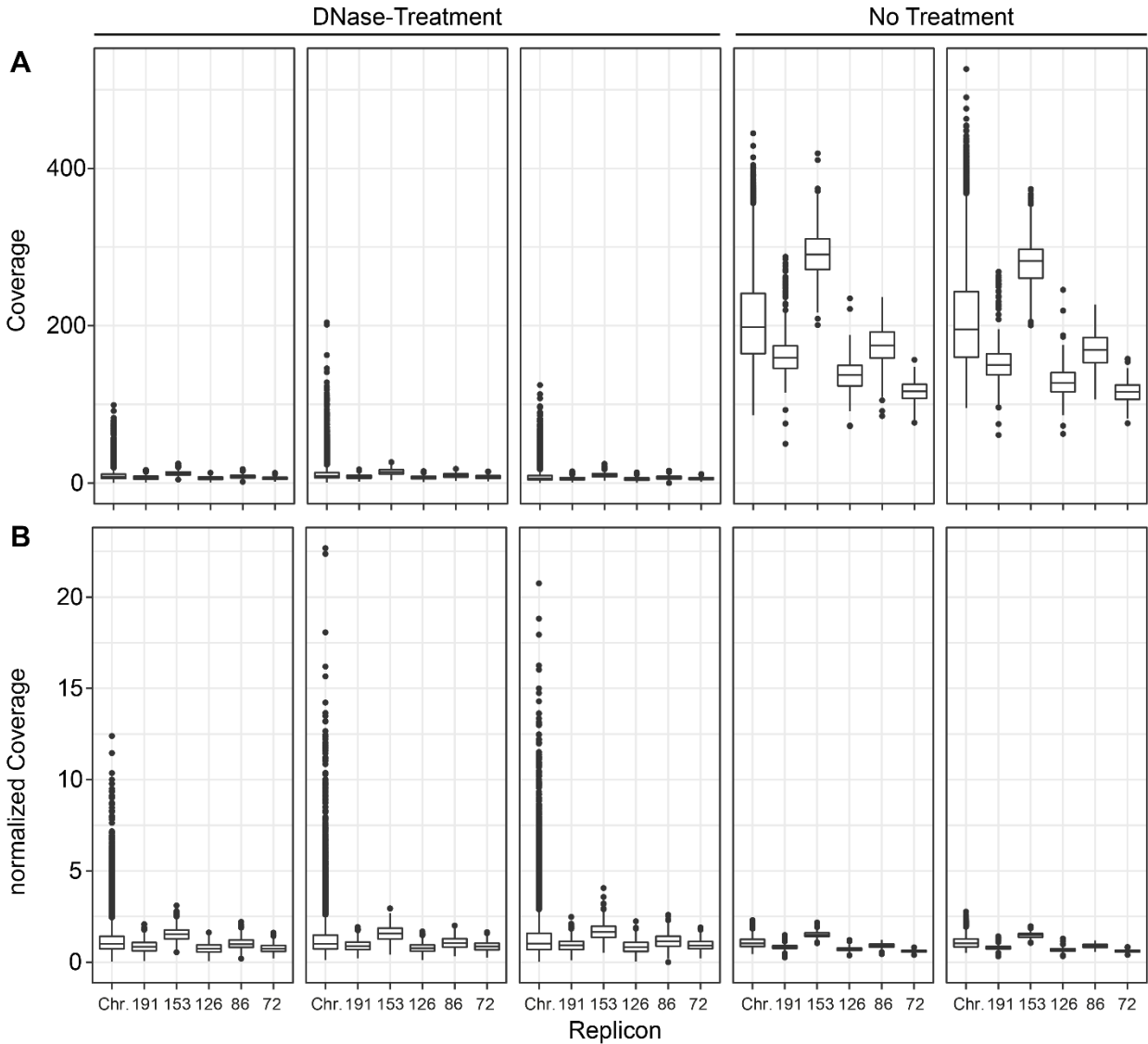

**Figure S3C**

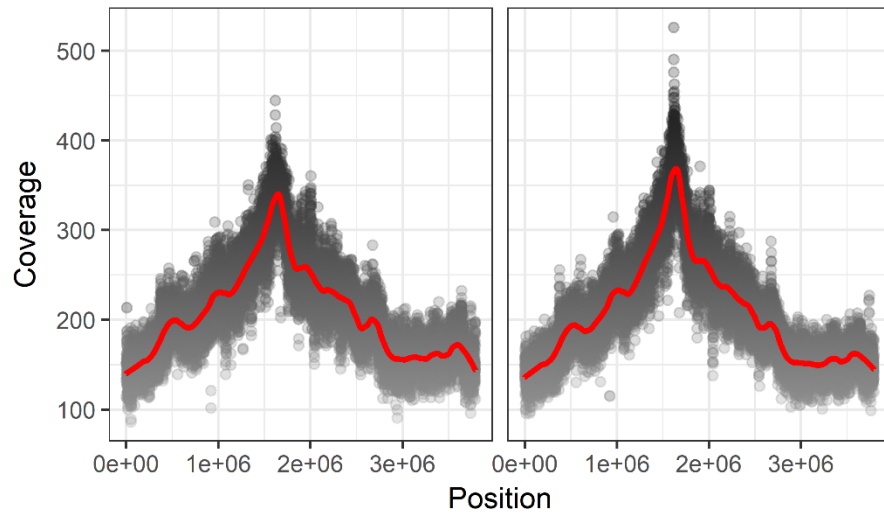

Supplement: FIG S3 [file mSystems.00693-20_sf003.pdf]
