# Supplementary material for: Dinoroseobacter shibae Outer Membrane Vesicles Are Enriched for the Chromosome Dimer Resolution Site dif
Source: mSystems. 2021 Jan 12;6(1):e00693-20. doi: 10.1128/mSystems.00693-20 (PMC7901474; doi:10.1128/mSystems.00693-20)

A

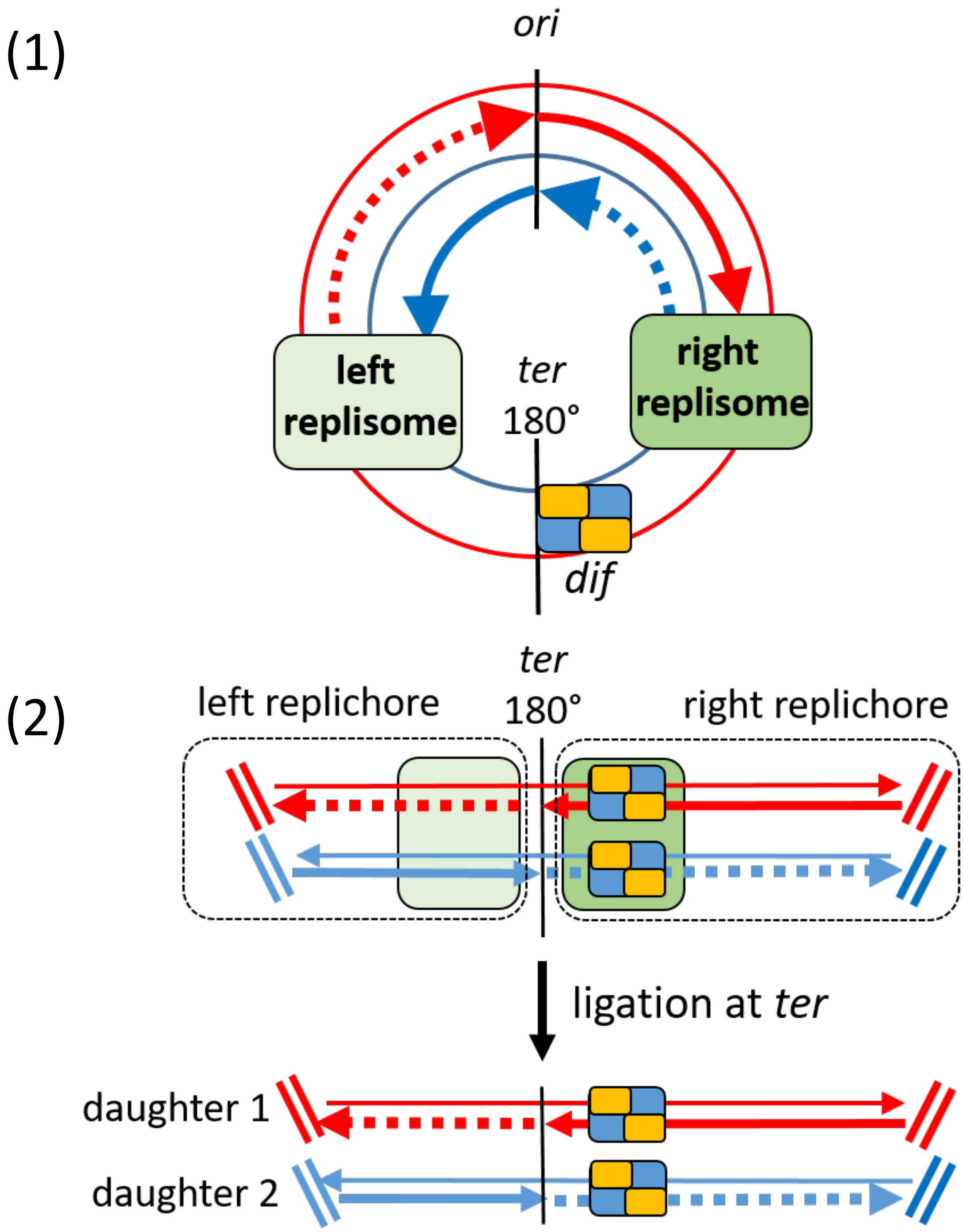
(1)
ori
left
replisome
ter
180°
right
replisome
dif
(2)
ter
180°
left replichore
right replichore
ligation at ter
daughter 1
daughter 2

B Overreplication Scenario 1: Strand Displacement & Switching

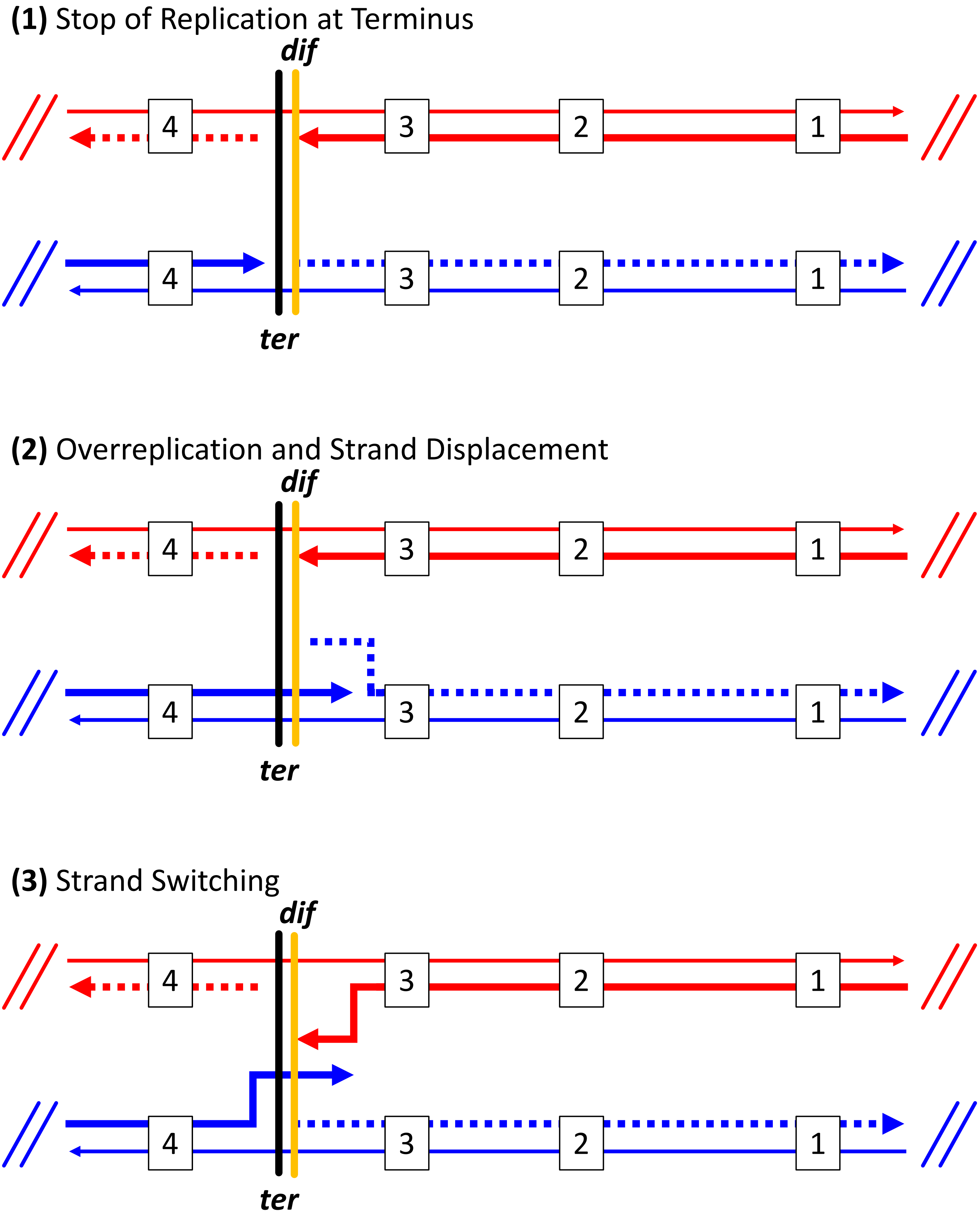

(4) Overreplication – Elongation Leading Strand (blue)

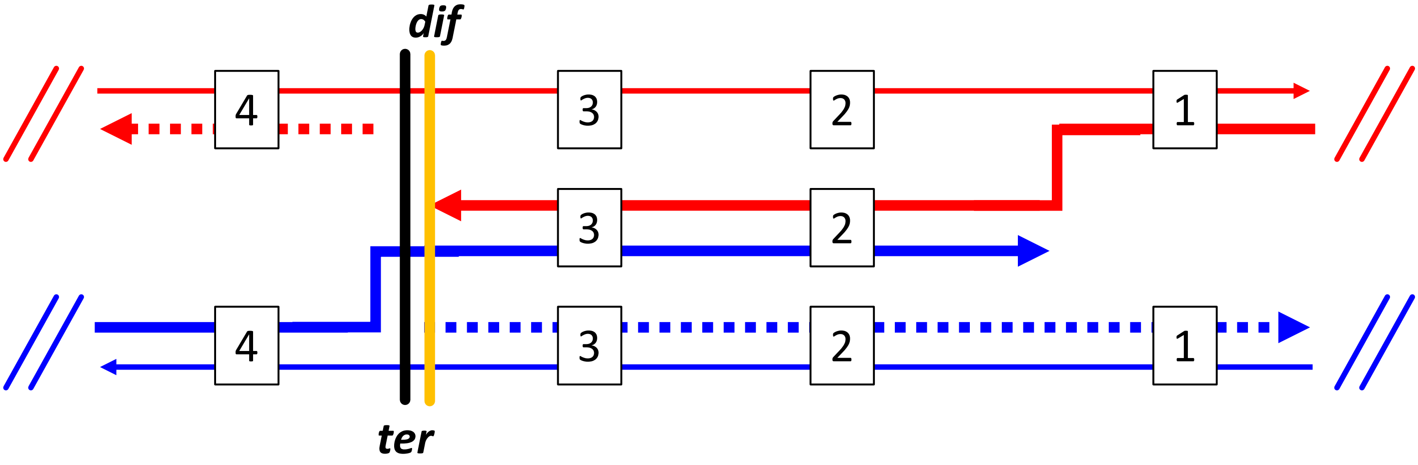

(5) Overreplication – Elongation Lagging Strand (red)

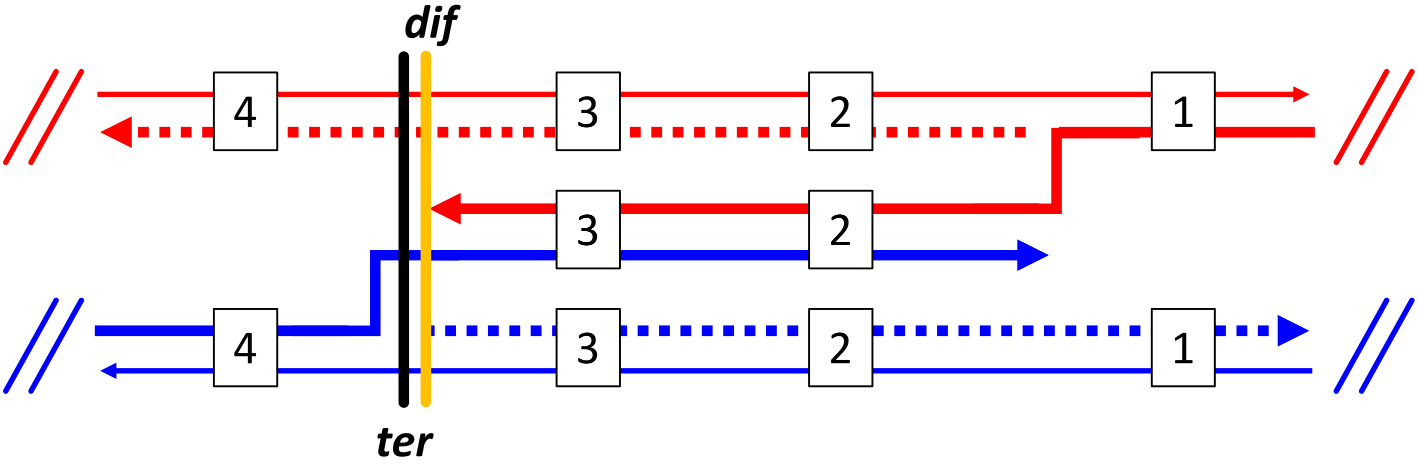

(6) Endonucleolytic Cleavage (single stranded DNA)

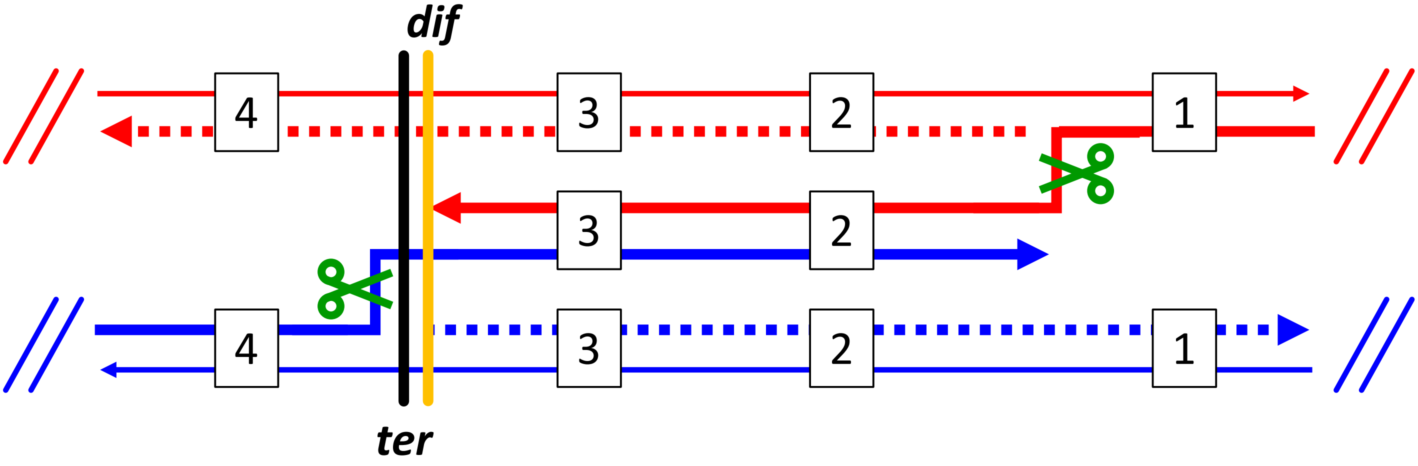

(7) Ligation of Leading & Lagging Strand

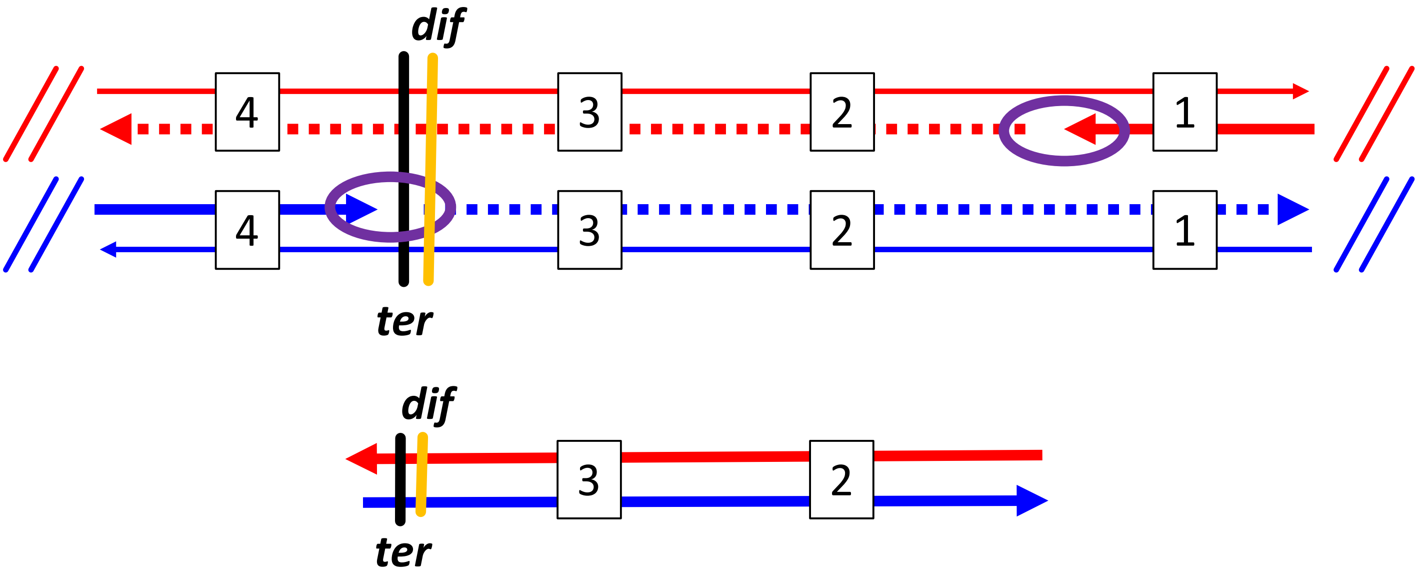

(8) Result: 2 Chromosomes & 1 linear Fragment

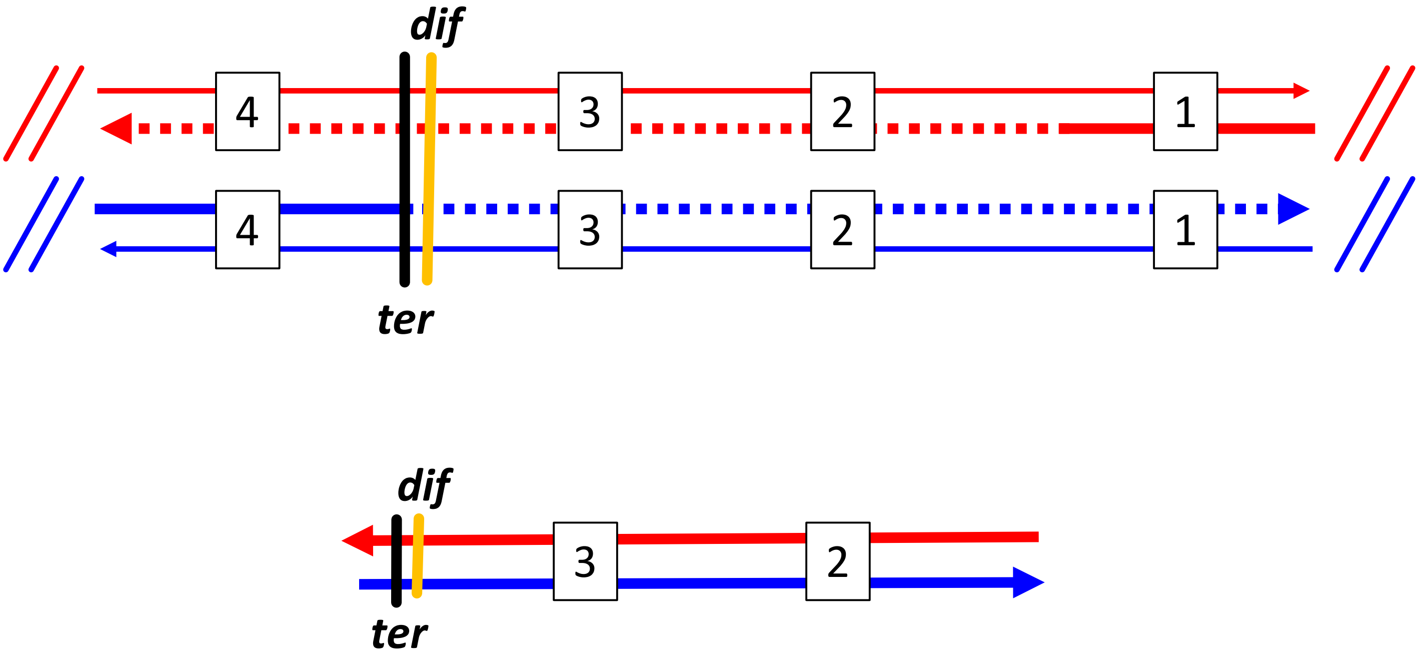

C Overreplication Scenario 2: Collision outside *ter*

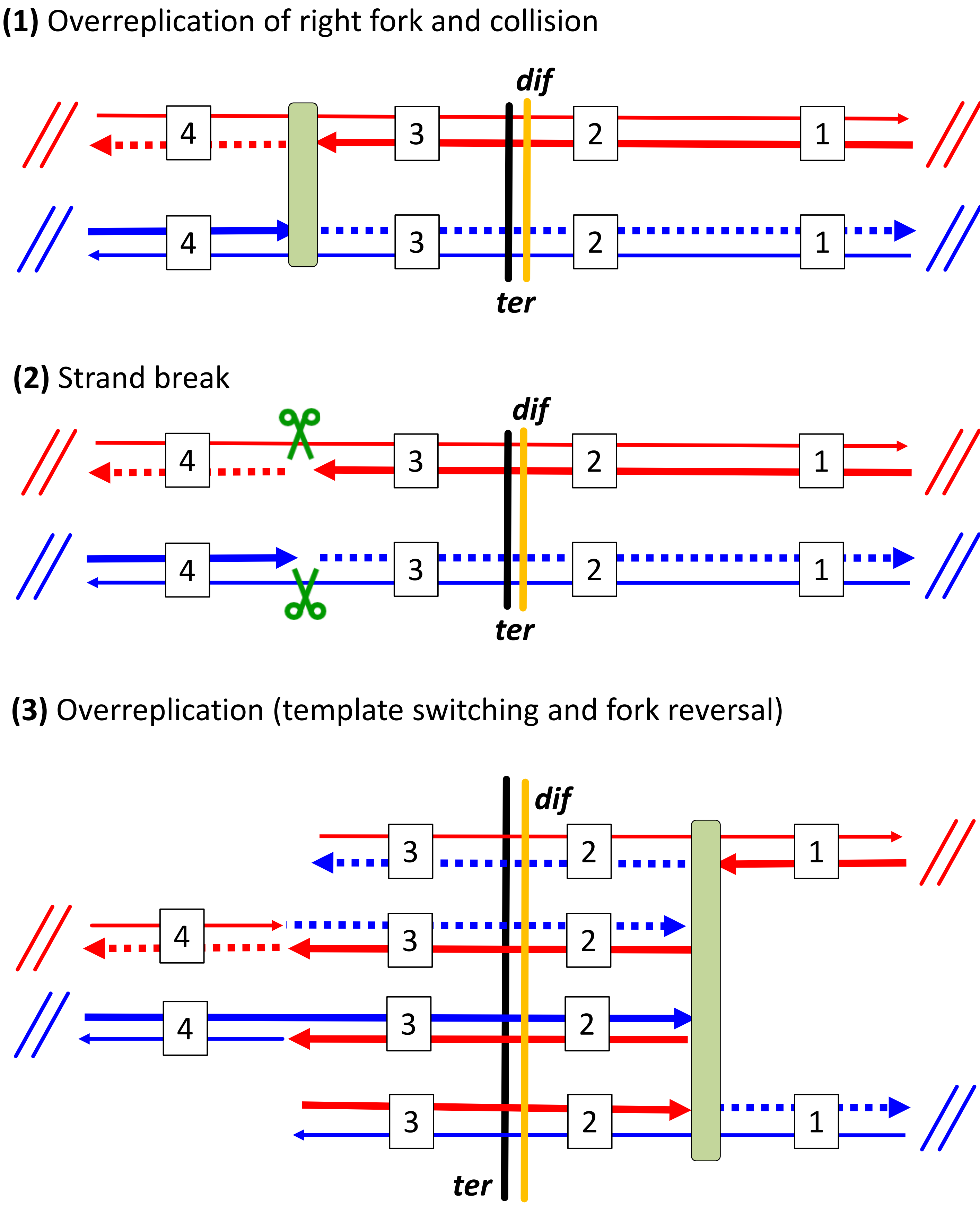

(4) Site specific recombination by XerCD (plus strand)

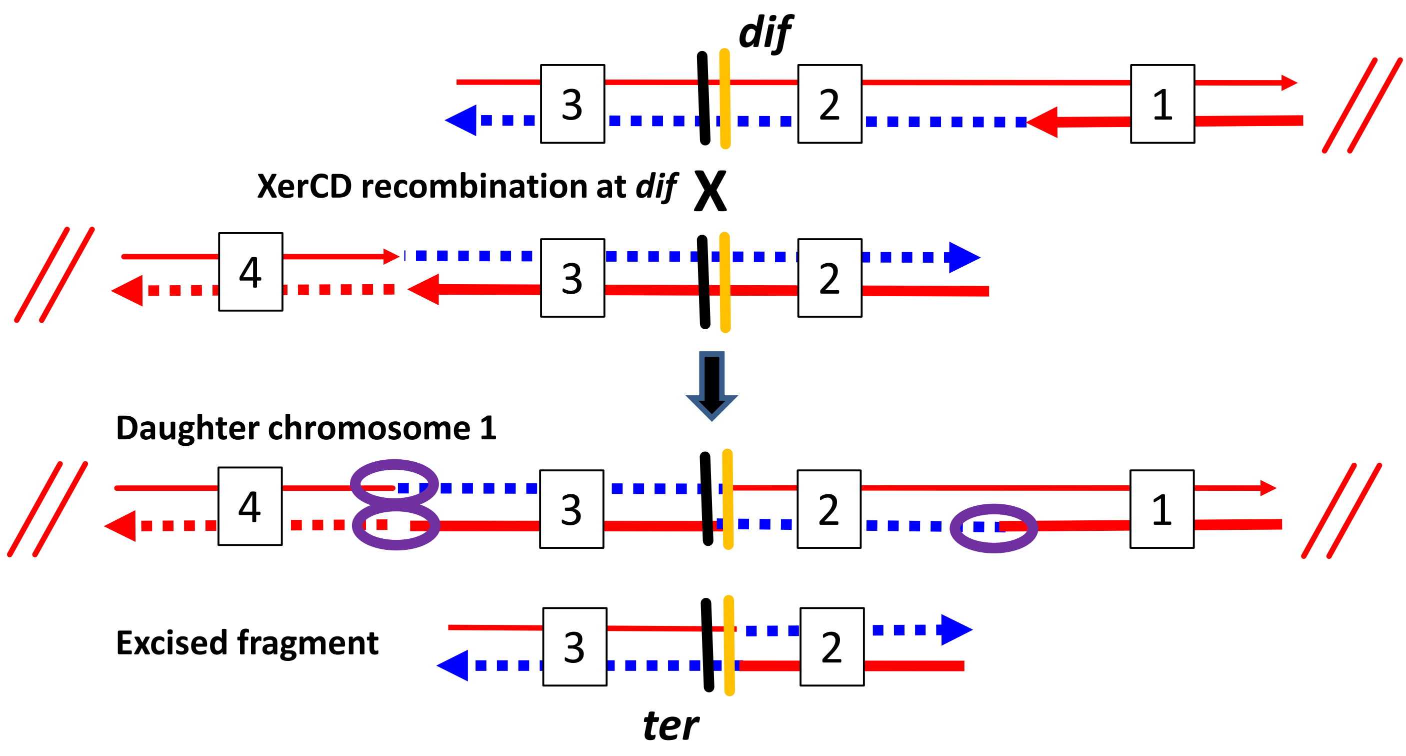

(5) Site specific recombination by XerCD (minus strand)

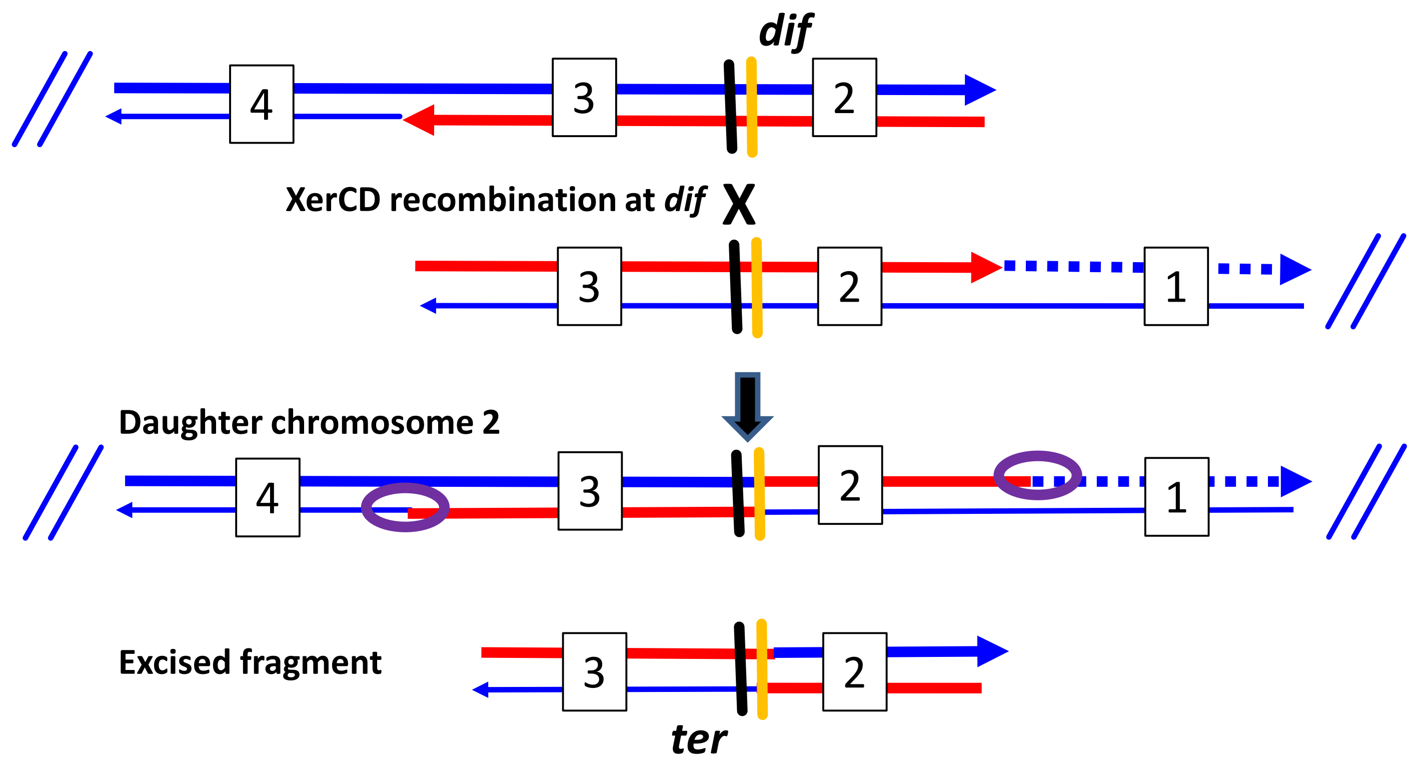

Supplement: FIG S4 [file mSystems.00693-20_sf004.pdf]
